# Supplementary material for: Impacts of body composition parameters and liver cirrhosis on the severity of alcoholic acute pancreatitis
Source: PLoS One. 2021 Nov 22;16(11):e0260309. doi: 10.1371/journal.pone.0260309 (PMC8608310; doi:10.1371/journal.pone.0260309)
Supplement: Attachment — Submitted filename: Response to reviewers comments_PONE-D-21-22616.doc [file pone.0260309.s002.doc]

**Responses to Reviewer’s Comments**

**Manuscript ID: PONE-D-21-22616**

**Title: Impacts of body composition parameters and liver cirrhosis on the severity of alcoholic acute pancreatitis**

**To Editor’s comments**

**Comment:** Your paper has been evaluated by two experts in the field (Reviewers #1 and #2) as well as a statistician (Reviewer #3). All have found merit in your study but think that the patients should be better characterized (e.g. how many had signs of chronic pancreatitis, whether only patients with the first episode of acute pancreatitis were involved in the analysis or whether patients had mild and moderate/severe acute pancreatitis).

**Response:** We appreciate your very constructive comments. We have sincerely answered the reviewers' comments below regarding your comment and revised the manuscript. In particular, we re-performed the overall statistical analysis with the help of a statistician (Pf. Sohee Oh) during the revision process, which was additionally mentioned in the acknowledgments. Most of the results did not change significantly, but the statistical significance of the MMA values in multivariable analysis decreased. This was thought to be the effect of clinical variables such as gender that are not expressed in the table. The changed results were reflected in the manuscript, and some expressions were also corrected (indicated in red color). We believe that the quality of this paper has been improved by the very sharp comments of all reviewers.

**To reviewer #1**

**Major Comment 1:** The methods sections needs to be extended and it should be described how mean muscle attenuation was detected and to what extent it indicates myosteatosis.

**Response:** The following sentence was added at the end of the method section, “CT-based body composition analysis”, with an additional reference.

The MMAs were measured by averaging CT attenuation of the abdominal muscles at the L3 level, including abdominal wall muscles, psoas muscles, quadratus lumborum muscles, and paraspinal muscles. The MMAs decrease as the amount of intramuscular fat, myosteatosis, increases [31].

**Major Comment 2:** It would be interesting to know, how many patients also had signs of chronic pancreatitis because chronic alcohol consumption not only predisposes to liver cirrhosis but also to chronic pancreatitis.

**Response:** We excluded both chronic alcohol drinkers and patients with chronic pancreatitis from the initial patient selection, considering the effect of chronic pancreatitis on outcomes. A total of 38 patients with chronic pancreatitis were excluded as indicated in Figure 1. This is more clearly stated in the methods as follows (changes are underlined).

All those with a high probability of AP due to other causes such as chronic pancreatitis, post-endoscopic retrograde cholangiopancreatography pancreatitis, periampullary cancer, intraductal papillary mucinous neoplasm, and autoimmune pancreatitis were excluded.

**Major Comment 3:** What was the Child-Pugh Score for liver cirrhosis in the patient group of mild vs. moderate/severe acute pancreatitis? It can be assumed that individuals with a lower muscle mass and moderate/severe acute pancreatitis more likely suffered from advanced stages of liver cirrhosis. These data should be incorporated into the manuscript.

**Response:** The result was already indicated in Table 4. When 32 patients with liver cirrhosis were divided into two groups (A and B/C) according to Child-Pugh class, the difference in the severity of acute pancreatitis was not significant, probably because the number of included patients with liver cirrhosis was small. This explanation was described in more detail as follows (changes are underlined).

*Methods, “Risk factors for moderately severe or severe alcoholic AP”*:

Characteristics of cirrhotic patients (N = 32) are described in Table 4. Clinical and body composition factors were not significantly different in the two groups. In particular, the difference in AAP severity according to Child-Pugh class was not significant.

*Discussion*:

Furthermore, AP patients with all etiologies were included in both studies. In contrast, we only included AAP patients, and in most liver cirrhosis was alcohol-induced. The fact that cirrhosis was found to well predict AP severity in our study probably stems from peripheral arterial vasodilation caused by systemic inflammation leading to organ failure [41]. Furthermore, concomitant alcoholic ketoacidosis or acute hepatitis may have affected outcomes. However, there was no significant difference in the AAP severity according to Child-Pugh class in our result, probably because there were small number of cirrhotic patients included.

**Minor Comment 1:** The authors should also mention the subdivision of patients in mild and moderate/severe acute pancreatitis in their methods section.

**Response:** We appreciate your attentive comments. The following sentence were added in the methods (study subjects) section.

The included AAP patients were divided into two groups according to AP severity (mild and moderately severe/severe).

**To reviewer #2**

**Comment 1:** It not known whether only patients with the first episode of acute pancreatitis were involved in the analysis. It is important since repeated episodes of acute alcoholic pancreatitis are quite common. The repeated episodes are causing irreversible changes in the pancreas, local complications occurs more frequently, and the pancreatitis can be more severe. This is a potential bias, therefore should be considered and patients with repeated attacks should be analyzed separately.

**Response:** We appreciate your sharp comment. Because we thought that recurrent episodes of acute pancreatitis would cause irreversible changes, as you mentioned, our study included only patients with first-time acute pancreatitis. This content was further described as follows (changes are underlined).

*Methods, Study subjects*:

The medical and radiographic records of adult patients with a diagnosis of first-time AP hospitalized at the Seoul Metropolitan Government Seoul National University Boramae Medical Center from 2011 to 2020 were identified in this retrospective study.

**Comment 2:** Line 58: outdated

**Response:** We have corrected the sentence as follows as per your point (changes are underlined).

Several scoring systems, such as the Ranson [11], APACHE-II [12], and BISAP [13] systems have been developed, but they are not widely used in practice because they are either outdated or complex.

**Comment 3:** Line 59: use laboratory parameters instead of hematological indicators

**Response:** We have corrected the sentence as follows as per your point (changes are underlined).

Researchers have tried to predict AP severity using various laboratory parameters such as C-reactive protein (CRP), blood urea nitrogen (BUN), and procalcitonin [14-16], but it has proven to be difficult to predict severity accurately using a single indicator.

**Comment 4:** Line 138: organ failure and local complications are defining the severity of pancreatitis, therefore it is obvious that they are occurring more frequently in the MSS group. These data are also shown in table one, the comparison of mild and MSS groups does not make much sense.

**Response:** As you mentioned, organ failure and local complications correspond to the definition of the severity of acute pancreatitis itself, so the expression in the results has been modified as follows.

Patients in the MSS group were significantly older, had a higher mean BUN level, and higher rates of liver cirrhosis~~, organ failure, and local complications~~.

However, even within the same severity group, the distribution of organ failure and local complications is different. Since Table 1 was intended to show the distribution of organ failure and local complications among patients with moderate to severe acute pancreatitis, the clinical characteristics of the included patients were reflected, so it was not deleted.

**To reviewer #3**

**Comment 1:** Can the authors provide a sample size/power paragraph, based on what effect sizes was envisioned (particularly, in the multiple logistic regression analysis)? The study recruited 242 patients with presumed AAP; however, some context on sample size/power would provide the reader a basis to understand how many subjects should be recruited in future studies of similar kinds. The sample size should be calculated, using the primary endpoint.

**Response:**

**Numeric Results ────────────────────────────────────────────────────────────**

**Pcnt N	Odds	R**

**Power	N	X=1	P0	P1	Ratio	Squared	Alpha	Beta**

**0.92574**	229	14	0.432	0.761	4.192	0.129	0.050	0.07426

**References**

Hsieh, F.Y., Block, D.A., and Larsen, M.D. 1998. 'A Simple Method of Sample Size Calculation for Linear and

Logistic Regression', Statistics in Medicine, Volume 17, pages 1623-1634.

**Report Definitions**

Power is the probability of rejecting a false null hypothesis. It should be close to one.

N is the size of the sample drawn from the population.

Pcnt N X=1 is the percentage of the population in which X = 1.

P0 is the response probability at the mean of X.

P1 is the response probability when X is increased to one standard deviation above the mean.

Odds Ratio is the odds ratio when P1 is on top. That is, it is [P1/(1-P1)]/[P0/(1-P0)].

R-Squared is the R2 achieved when X is regressed on the other independent variables in the regression.

Alpha is the probability of rejecting a true null hypothesis.

Beta is the probability of accepting a false null hypothesis.

**Summary Statements ─────────────────────────────────────────────────────────**

A logistic regression of a binary response variable (Y) on a binary independent variable (X)

with a sample size of 229 observations (of which 86% are in the group X=0 and 14% are in the

group X=1) achieves 93% power at a 0.050 significance level to detect a change in Prob(Y=1)

from the baseline value of 0.432 to 0.761. This change corresponds to an odds ratio of 4.192.

An adjustment was made since a multiple regression of the independent variable of interest on

the other independent variables in the logistic regression obtained an R-Squared of 0.129.

**Comment 2:** The multiple logistic regressions should be followed by a desired goodness-of-fit assessment, say, via the Hosmer-Lemeshow statistic. This is missing in this submission.

**Response:** The following result was added in the table 3. “Hosmer-Lemeshow goodness of fit test (multiple logistic regression analysis), *P* value = 0.1574.”

**Comment 3:** The authors should make sure that explanation of covariate effects in the Results section should always be followed with the estimate, and respective 95% interval.

**Response:** 95% CIs were added in the result and discussion as indicated in red color.

**Comment 4:** The Discussion should allude to future studies conducted on a larger scale on subjects recruited at other countries to better understand the relationships between AP severity, and body composition parameters or liver cirrhosis.

**Response:** The following sentence was added in the last paragraph of discussion. “A large-scale prospective study is also needed to better understand the relationship between AAP severity and body composition parameters or liver cirrhosis.”

We would like to thank once again for the excellent comments that significantly contributed to the quality of the manuscript. We hope that this revised manuscript will be acceptable for publication in ***PLOS ONE.***
